# Characteristics and outcomes of patients with severe COVID-19 in Indonesia: Lessons from the first wave

**DOI:** 10.1371/journal.pone.0290964

**Published:** 2023-09-25

**Authors:** Erlina Burhan, Keibun Liu, Eva M. Marwali, Samuel Huth, Navy G. H. M. L. Wulung, Dafsah A. Juzar, Muhammad A. Taufik, Surya O. Wijaya, Dyah K. Wati, Neurinda P. Kusumastuti, Saptadi Yuliarto, Bhirowo Y. Pratomo, Erwin Pradian, Dadang H. Somasetia, Desy Rusmawatiningtyas, Arie Z. Fatoni, Jose M. Mandei, Eka Y. Lantang, Fajar Perdhana, Bambang P. Semedi, Muhammad Rayhan, Tiffany R. S. Tarigan, Nicole White, Gianluigi L. Bassi, Jacky Y. Suen, John F. Fraser

**Affiliations:** 1 Faculty of Medicine, Department of Pulmonology and Respiratory Medicine, Universitas Indonesia and Persahabatan Hospital, Jakarta, Indonesia; 2 Critical Care Research Group, The Prince Charles Hospital, Brisbane, QLD, Australia; 3 Faculty of Medicine, The University of Queensland, Brisbane, QLD, Australia; 4 Pediatric Cardiac Intensive Care Unit, National Cardiovascular Center Harapan Kita, Jakarta, Indonesia; 5 Intensive Care Unit, Persahabatan Hospital, Jakarta, Indonesia; 6 Departement of Cardiology and Vascular Medicine, Intensive Cardiovascular Care Unit, National Cardiovascular Center Harapan Kita and Universitas Indonesia, Jakarta, Indonesia; 7 Anesthesiology and Critical Care Department, Fatmawati General Hospital, Jakarta, Indonesia; 8 Intensive Care Unit, Sulianti Saroso Hospital, Jakarta, Indonesia; 9 Pediatric Intensive Care Unit, Sanglah Hospital, Denpasar, Bali, Indonesia; 10 Pediatric Intensive Care Unit, Universitas Airlangga Hospital, Surabaya, East Java, Indonesia; 11 Pediatric Intensive Care Unit, Saiful Anwar Hospital, Malang, East Java, Indonesia; 12 Intensive Care Unit, Sardjito Hospital, Jogya, Indonesia; 13 Intensive Care Unit, Hasan Sadikin Hospital, Bandung, West Java, Indonesia; 14 Pediatric Intensive Care Unit, Hasan Sadikin Hospital, Bandung, West Java, Indonesia; 15 Pediatric Intensive Care Unit, Sardjito, Yogyakarta, Indonesia; 16 Intensive Care Unit, Saiful Anwar Hospital, Malang, East Java, Indonesia; 17 Pediatric Intensive Care Unit, RSUP Prof Dr R. D. Kandou Manado, Indonesia; 18 Intensive Care Unit, RSUP Prof Dr R. D. Kandou Manado, Indonesia; 19 Intensive Care Unit, Universitas Airlangga Hospital, Surabaya, East Java, Indonesia; 20 Intensive Care Unit, RSUD Dr Soetomo Surabaya, East Java, Indonesia; Azienda Ospedaliero Universitaria Careggi, ITALY

## Abstract

**Background:**

Indonesia’s national response to COVID-19 evolved rapidly throughout 2020. Understanding pandemic response and outcomes is crucial for better mitigation strategies ahead. This study describes the characteristics and outcomes of patients admitted to ICU during the early stages of the pandemic.

**Methods:**

This is a multi-centre prospective observational study including patients from twelve collaborating hospitals in Indonesia. All patients were clinically suspected or laboratory-confirmed COVID-19 cases admitted to ICU between January 2020 and March 2021. The primary outcome was monthly ICU mortality. Descriptive statistics of patient characteristics and treatment were generated as secondary outcomes.

**Results:**

From 559 subjects, the overall mortality was 68% and decreased over the study period, while the mortality of patients that received mechanical ventilation was 92%, consistently high over the study period. Fatal cases showed 2- and 4-day delays from symptoms onset to hospital admissions and ICU admissions, respectively. Evidence-backed approaches which could influence patient outcome, such as extracorporeal membrane oxygenation, prone positioning, renal replacement therapy, and neuromuscular blockade were scarcely administered.

**Conclusions:**

The mortality rate of COVID-19 patients in Indonesia was extremely high during the first major outbreak of disease, particularly in those mechanically ventilated. Delayed admission and unavailability of evidence-based approaches due to high burden on health facility during COVID-19 crisis could be addressed by efficient public health measures and enhancing health infrastructure to improve the future pandemic response.

## Introduction

The Coronavirus Disease 2019 (COVID-19) pandemic has had a devastating impact on global health with over 5 million deaths over the past two years. Although the COVID-19 vaccination has stunted the spread and mortality of the disease, the number of new cases and death remains high [1,2]. The continuing impact of the COVID-19 pandemic on each country is not homogenous because of differences in healthcare systems, border and infectious control policy, and available resources [3–5]. The COVID-19 impacts can be severe and long-lasting for developing countries with limited resource [2,6]. Understanding this inhomogeneity necessitates the reporting of characteristics and outcomes of patients with COVID-19 across multiple regions [7]. Such reporting benefits clinical guidance in the country and provides important insights to other countries.

Indonesia is geographically distinct as a collection of small remote islands with a large population of 272 million people. While geographic isolation can be protective with tight border control, population density conveys weakness to the rapid spread of infection within the country, which could result in worse outcomes [8–10]. In the early pandemic stage, health systems were largely unprepared. As of April 2020, there were only 48 laboratories assigned by the Ministry of Health across the country for COVID-19 diagnostics. Citizens in the remote and disadvantaged Indonesian regions were more vulnerable, as the access to health service remains limited [11]. Understanding the response and outcomes during this early stage is crucial for better mitigation strategies in the future. However, a national overview of the patients with COVID-19 infection who were critically ill and admitted to the Intensive Care Unit (ICU) is lacking. While aggregate statistics of infections and deaths are publicly available, no studies have described the patient and treatment characteristics of Indonesian COVID-19 patients.

Therefore, we performed a prospective observational study in collaboration with multiple centers in Indonesia that have treated critically ill patients with COVID-19.

## Materials and methods

This is a multi-centre prospective observational study with seven hospitals in Indonesia. This study is a sub-analysis of a large-scale international registry (COVID-19 Critical Care Consortium) that aims to collect the data of ICU patients with COVID-19 infections in collaborations with 388 participating hospitals across 54 countries in the world [12,13]. Data gathered by the collaborating investigators in large tertiary hospitals in Indonesia was used in this study. The full study protocol of the international registry, data entry list with each definition, and instructions on how to complete the case report form are all available online (https://www.elso.org/COVID-19/ECMOCARD.aspx). The ethics committee at each hospital approved the study and confirmed the waivers of informed consent because of the nature of de-identified data. This study was registered in Indonesian Ministry of Health Ethical Board with registered number LB 02.01/ 2/ KE 418/ 2020.

Throughout the study period, patients were enrolled consecutively if they had clinically suspected or laboratory-confirmed SARS-CoV-2 infection and were admitted to the ICU. Clinical suspicion is based on the national COVID-19 guideline, for patients with clinical symptoms and without confirmed positive PCR result. All study site followed the national guideline from the Ministry of Health [14]. Patients were excluded if they lacked core variables pertaining to key dates (hospital admission, ICU admission, death/discharge) or outcome.

### Data collection

#### Baseline characteristics of the patients

Age, gender, body mass index, comorbidities, symptoms at the time of hospital admission, and number of patients who had multi-symptoms, were identified in Table 1.

**Table 1:** Baseline characteristics.

Variable	All patients (n = 559)	Survivors (n = 181)	Deaths (n = 378)	P-Value	Mechanical Ventilation		
					Survivors (n = 28)	Deaths (n = 302)	P- Value
Age (years), median [IQR]	55 (45–64)	51 (35–60)	57 (47–65)	<0.001	**36 (26–53)**	55 (45–64)	<0.001
Gender (male), n (%)	360 (64%)	112 (62%)	248 (66%)	0.4	**13 (46%)**	360 (64%)	0.036
BMI (kg/m2), median [IQR]	24.7 (22.4–28.0)	25.1 (22.5–28.3)	24.5 (22.3–27.7)	0.4	24.5 (22.2–27.7)	24.7 (22.4–28.0)	0.9
**Comorbidities at hospital admission, n (%)**							
Hypertension	234 (42%)	73 (41%)	161 (43%)	0.014	**5 (18%)**	**125 (42%)**	0.014
Diabetes	168 (31%)	48 (27%)	120 (32%)	0.2	**4 (15%)**	**96 (32%)**	0.057
Smoking	103 (18%)	31 (17%)	72 (19%)	0.4	4 (14%)	63 (21%)	0.09
Obesity	110 (20%)	42 (23%)	68 (19%)	0.9	5 (18%)	56 (19%)	0.9
Chronic cardiac disease	179 (32%)	78 (43%)	101 (27%)	<0.001	5 (18%)	69 (23%)	0.5
**Symptoms at hospital admission, n (%)**							
Dyspnea	472 (84%)	132 (73%)	340 (90%)	<0.001	22 (79%)	275 (91%)	0.12
Fever	350 (63%)	97 (54%)	253 (67%)	<0.001	17 (61%)	214 (71%)	0.3
Cough	374 (67%)	106 (59%)	263 (72%)	0.002	18 (64%)	214 (71%)	0.3
Fatigue / Malaise	248 (44%)	74 (41%)	174 (46%)	0.3	7 (25%)	151 (50%)	0.027
Vomiting / Nausea	165 (30%)	44 (24%)	121 (32%)	0.093	10 (36%)	102 (34%)	0.9
Sore throat	88 (16%)	20 (11%)	68 (18%)	0.012	4 (14%)	63 (21%)	0.7
Headache	70 (13%)	15 (8%)	55 (15%)	0.036	5 (18%)	48 (16%)	0.8
Chest pain	86 (15%)	44 (24%)	42 (11%)	<0.001	4 (14%)	32 (11%)	0.6
Diarrhoea	47 (8.4%)	16 (9%)	31 (8.8%)	0.7	4 (14%)	26 (9%)	0.4
Altered consciousness / confusion	62 (11%)	6 (3.3%)	56 (15%)	<0.001	2 (7%)	48 (16%)	0.4
Runny nose	45 (8.1%)	12 (6.6%)	33 (8.7%)	0.022	2 (7%)	32 (11%)	0.6
Loss of taste	22 (4.0%)	5 (3%)	17 (4.5%)	0.061	1 (4%)	17 (6%)	0.2
**Number of patients who had dyspnea, fever, cough, and fatigue/malaise, n (%)**	124 (22%)	**26 (14%)**	**98 (25%)**	0.002	**5 (18%)**	**86 (28%)**	0.23

IQR interquartile range, BMI body mass index.

#### Treatment

The type of medical supports that patients received, such as non-invasive mechanical ventilation, high flow nasal cannula, invasive mechanical ventilation, use of inotropes, tracheostomy, extracorporeal membrane oxygenation (ECMO), prone positioning, renal replacement therapy, and neuromuscular blockade, and the use of antiviral agents, potential drugs to treat COVID-19 infection, corticosteroid, and anticoagulation agents), were mentioned in Table 2.

**Table 2:** Treatments.

Variable	All patients (n = 559)	Survivors (n = 181)	Deaths (n = 378)	Mechanical Ventilation	
				Survivors (n = 28)	Deaths (n = 302)
Medical Support, n (%)					
Oxygen therapy	466 (84%)	160 (88%)	306 (81%)	26 (93%)	235 (78%)
Non−invasive ventilation	78 (14%)	31 (17%)	47 (12%)	10 (36%)	24 (8.7%)
** High flow nasal cannula oxygen**	**40 (35%)**	**4 (14%)**	**35 (47%)**	1 (33%)	21 (49%)
** Invasive mechanical ventilation**	**330 (59%)**	**28 (15%)**	**286 (81%)**	---	---
** Inotropes / vasopressors**	**324 (58%)**	**38 (21%)**	**286 (76%)**	**18 (64%)**	**263 (87%)**
Tracheostomy	9 (1.6%)	0 (0%)	9 (2.3%)	0 (0%)	9 (2.9%)
ECMO	7 (1.3%)	**1 (0.5%)**	**6 (1.6%)**	**1 (3.6%)**	**6 (2.0%)**
** Prone positioning**	**30 (5.4%)**	**10 (5.5%)**	**20 (5.5%)**	**2 (7.4%)**	**18 (6.0%)**
Inhaled Nitric Oxide	8 (1.4%)	3 (1.7%)	5 (1.3%)	**2 (7.1%)**	**5 (1.6%)**
Renal replacement therapy	60 (11%)	4 (2.2%)	56 (15%)	**3 (11%)**	**54 (18%)**
** Neuromuscular blockade**	**94 (17%)**	**8 (4.5%)**	**86 (23%)**	**8 (29%)**	**85 (28%)**
Prescription, n (%)					
Antivirals, n (%)					
Ribavirin	0 (0%)	0 (0%)	0 (0%)	0 (0%)	0 (0%)
Lopinavir ritonavir	3 (0.5%)	0 (0%)	3 (0.8%)	0 (0%)	3 (1.0%)
Remdesivir (Veklury)	43 (7.7%)	10 (5.5%)	33 (8.7%)	1 (3.6%)	25 (8.3%)
Interferon alpha	1 (0.2%)	0 (0%)	1 (0.3%)	0 (0%)	1 (0.3%)
Interferon beta	0 (0%)	0 (0%)	0 (0%)	0 (0%)	0 (0%)
Hydroxychloroquine	123 (22%)	39 (22%)	84 (22%)	5 (18%)	69 (22%)
Interleukin-6 (IL-6) inhibitor	1 (0.1%)	0 (0%)	1 (0.3%)	0 (0%)	1 (0.5%)
Convalescent plasma	1 (0.1%)	0 (0%)	1 (0.3%)	0 (0%)	1 (0.5%)
** Oseltamivir**	**299 (53%)**	**103 (57%)**	**196 (51%)**	20 (71%)	165 (53%)
Use of corticosteroid, n (%)					
Dexamethasone (6mg/day)	21 (3.8%)	**0 (0%)**	**21 (5.6%)**	**0 (0%)**	**14 (4.6%)**
Dexamethasone (other dosage)	13 (2.3%)	**3 (1.7%)**	**10 (2.6%)**	**2 (7.1%)**	**5 (1.7%)**
Other than dexamethasone	11 (2.0%)	**0 (0%)**	**11 (3.1%)**	**0 (0%)**	**6 (1.8%)**
Anticoagulation therapy, n (%)					
Therapeutic treatment of DVT/PE	21 (3.8%)	**6 (3.3%)**	**15 (4.0%)**	**1 (3.6%)**	**7 (2.3%)**
Prophylaxis for COVID-19	28 (5.0%)	**2 (1.1%)**	**26 (6.9%)**	**0 (0%)**	**18 (6.0%)**
Anticoagulation agent					
Unfractionated heparin	19 (3.6%)	3 (1.6%)	16 (4.6%)	1 (50%)	10 (3.9%)
Low molecular weight heparin	29 (5.2%)	5 (2.7%)	24 (6.4%)	0 (0%)	15 (5.1%)
Enoxaparin	9 (1.9%)	1 (1.1%)	8 (2.3%)	1 (50%)	5 (1.6%)
Warfarin	7 (1.2%)	1 (0.5%)	6 (1.5%)	0 (0%)	2 (0.6%)
Others	1 (0.1%)	0 (0%)	1 (0.3%)	0 (0%)	1 (0.3%)

ECMO extracorporeal membrane oxygenation.

#### Clinical parameters

We evaluated the changes in the clinical parameters as a daily basis (i.e., vital signs, including Glasgow Coma Score, heart rate, mean arterial pressure, respiratory rate, and temperature, and laboratory data, for example, white blood cell, lymphocyte cell, neutrophil cell, platelet, and c-reactive protein.

#### Complications

Bacterial pneumonia, bacteremia, pneumothorax, myocardial infarction, acute kidney injury, gastrointestinal hemorrhage, hyper or hypoglycemia, etc, were mentioned in Table 3.

**Table 3:** Complications during ICU stay.

Variable	All patients (n = 559)	Survivors (n = 181)	Deaths (n = 378)	Mechanical Ventilation	
				Survivors (n = 28)	Deaths (n = 302)
Number of patients who had at least one complication during ICU stay, n (%)	408 (72%)	103 (56%)	307 (79%)	13 (46%)	252 (81%)
Infectious complications, n (%)					
**Bacterial pneumonia**	**116 (21%)**	**16 (9%)**	**100 (27%)**	8 (30%)	92 (31%)
**Bacteraemia**	**55 (10%)**	**6 (3.3%)**	**49 (13%)**	**2 (7%)**	**44 (15%)**
Other complications, n (%)					
Pneumothorax	11 (2%)	0 (0%)	11 (2.9%)	0 (0%)	10 (3.3%)
Myocardial infraction	35 (6.4%)	10 (5.5%)	25 (6.8%)	1 (4%)	19 (6.4%)
Cardiac arrhythmia	79 (14%)	21 (12%)	58 (15%)	2 (7%)	46 (15%)
Myocarditis/Pericarditis	39 (7.1%)	13 (7%)	26 (7%)	2 (7%)	21 (7%)
Endocarditis	2 (0.4%)	2 (1%)	0 (0%)	1 (4%)	0 (0%)
Cardiomyopathy	12 (2.2%)	7 (4%)	5 (1%)	1 (4%)	3 (1%)
Congestive heart failure	102 (18%)	46 (25%)	56 (15%)	0 (0%)	34 (11%)
Seizure	9 (1.6%)	2 (1%)	7 (2%)	1 (4%)	6 (2%)
Stroke	14 (2.5%)	2 (1%)	12 (3%)	0 (0%)	7 (2.3%)
Meningitis/Encephalitis	3 (0.5%)	0 (0%)	3 (1%)	0 (0%)	2 (1%)
Pulmonary embolism	10 (1.8%)	1 (1%)	9 (2%)	0 (0%)	8 (3%)
Deep vein thrombosis	6 (1%)	0 (0%)	6 (2%)	0 (0%)	4 (2%)
Acute renal injury	237 (43%)	**46 (25%)**	**191 (51%)**	**8 (29%)**	**161 (53%)**
Gastrointestinal haemorrhage	69 (12%)	**13 (7%)**	**56 (15%)**	5 (18%)	45 (15%)
Pancreatitis	0 (0%)	0 (0%)	0 (0%)	0 (0%)	0 (0%)
Hyperglycaemia	178 (32%)	**36 (20%)**	**142 (38%)**	**5 (18%)**	**120 (40%)**
Hypoglycaemia	49 (8.8%)	**9 (5%)**	**40 (10%)**	**0 (0%)**	**34 (11%)**

ICU; intensive care unit.

#### Outcomes

Time from symptom onset to hospital admission, ICU admission, ICU discharge, and hospital discharge, mechanical ventilation period, length of ICU and hospital stay, in-hospital mortality, 28-day mortality, a primary cause of death, and discharge destination. The primary outcome of this study was in-hospital mortality were mentioned in Table 4.

**Table 4:** Other outcomes.

Variable	All patients (n = 559)	Survivors (n = 181)	Deaths (n = 378)	Mechanical Ventilation	
				Survivors (n = 28)	Deaths (n = 302)
Days from hospital admission to initiate antibiotics treatment, median [IQR]	0 (0–1)	0 (0–2)	0 (0–1)	0 (0–2)	0 (0–1)
Days of use of corticosteroid, median [IQR]	6 (4–10)	7 (5–10)	5 (3–9)	6 (5–8)	6 (4–9)
Mechanical ventilation period (days), median [IQR]	4 (2–8)	5 (3–9)	4 (2–8)	5 (3–9)	4 (2–8)
ICU Length of stay (days), median [IQR]	6 (2–10)	10 (5–15)	4 (2–8)	11 (7–17)	5 (2–9)
Hospital Length of stay (days), median [IQR]	8 (4–15)	13 (10–22)	6 (3–11)	18 (12–28)	6 (3–11)
Cause of death, n (n)					
Respiratory failure	157 (42%)	---	157 (42%)	---	121 (40%)
Multi-organ failure	73 (19%)	---	73 (19%)	---	51 (17%)
Septic Shock	70 (18%)	---	70 (18%)	---	66 (22%)
Not reported	29 (8%)	---	29 (8%)	---	31 (10%)
Cardiac Failure	29 (8%)	---	29 (8%)	---	21 (7%)
Other	13 (3%)	---	13 (3%)	---	10 (3%)
Haemorrhagic shock	3 (1%)	---	3 (1%)	---	2 (1%)
Cardiovascular accident	2 (1%)	---	2 (1%)	---	2 (1%)
Cerebrovascular accident	1 (0%)	---	1 (0%)	---	0 (0%)

IQR interquartile range, ICU intensive care unit.

After assessing the distribution by Kolmogorov–Smirnov testing, normally distributed continuous data were described as mean with standard deviations (SD) and non-normally distributed continuous data as medians with interquartile range (IQR). The categorical data including dichotomous and ordinal variable were displayed as numbers and/or percentages for each category. The monthly mortality was depicted by the hospital admission date of the patient. The clinical parameters of vital sign and laboratory data were plotted across days of ICU stay. This study is primarily descriptive with limited comparison of groups stratified by mortality or other characteristics. The objective is to generate insights and hypotheses for subsequent study where appropriate. Patients who underwent mechanical ventilation during their ICU stay were separately demonstrated to highlight this important sub-population. All statistical analyses were performed with the use of R (R version 4.0.3, R Project, Vienna, Austria).

## Results

### Mortality of the patients with COVID-19 infection in Indonesia

**Fig 1:**
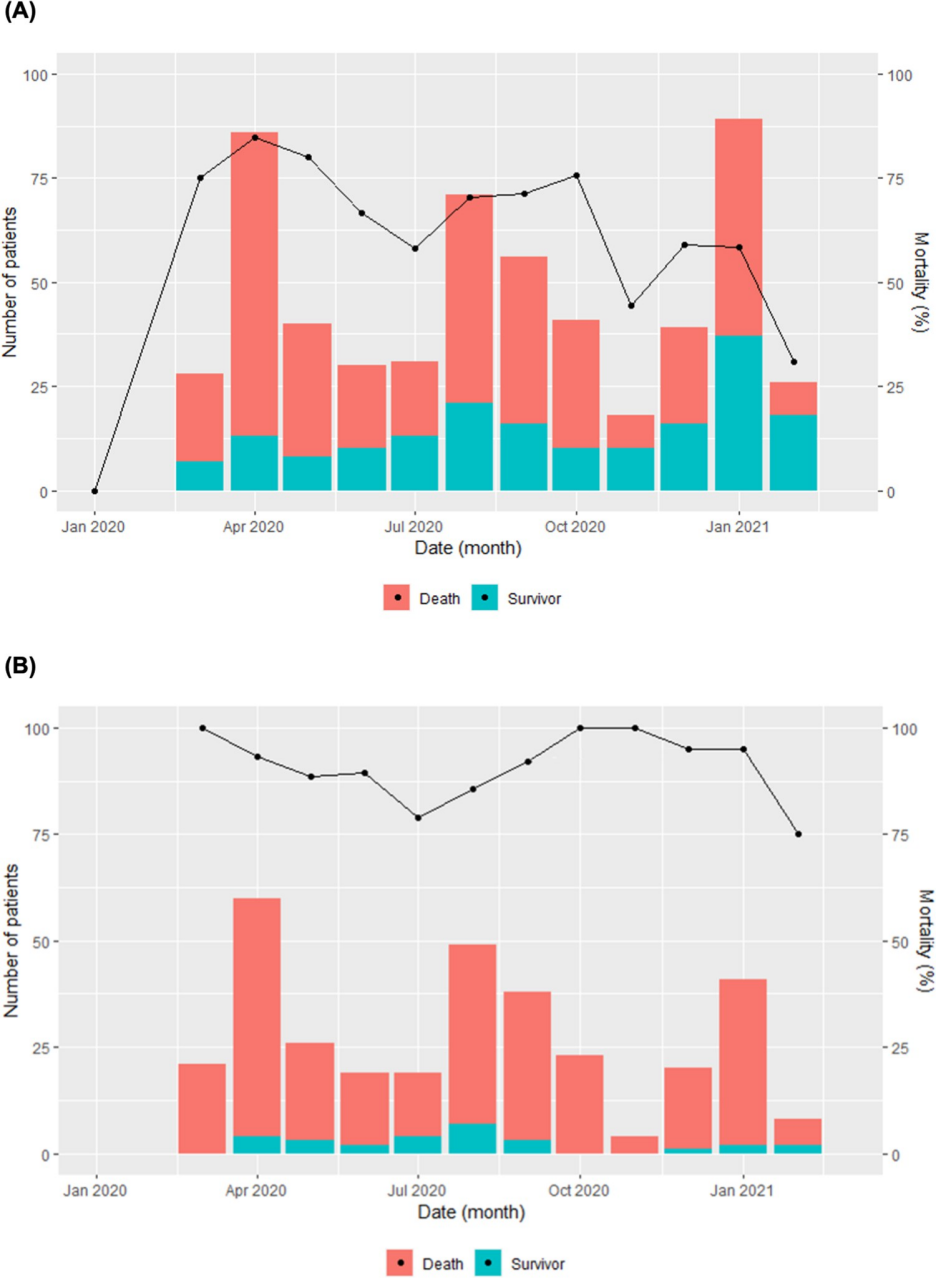
Number of cases and their outcome by month. (A) Overall in-hospital mortality. (B) In-hospital mortality of mechanically ventilated patients; The bar graphs show the number of cases, and the line graphs show mortality (percentage).

A total of 559 patients were enrolled in this study after excluding 291 patients with missing data on survival or death. The overall in-hospital mortality was 68%, especially high in those receiving mechanical ventilation, numbering 92%. Overall, in-hospital mortality decreased across the study period, while the in-hospital mortality of mechanically ventilated patients was consistently high (Fig 1).

### Differences in the time course of the progression of COVID-19 infection

**Fig 2:**
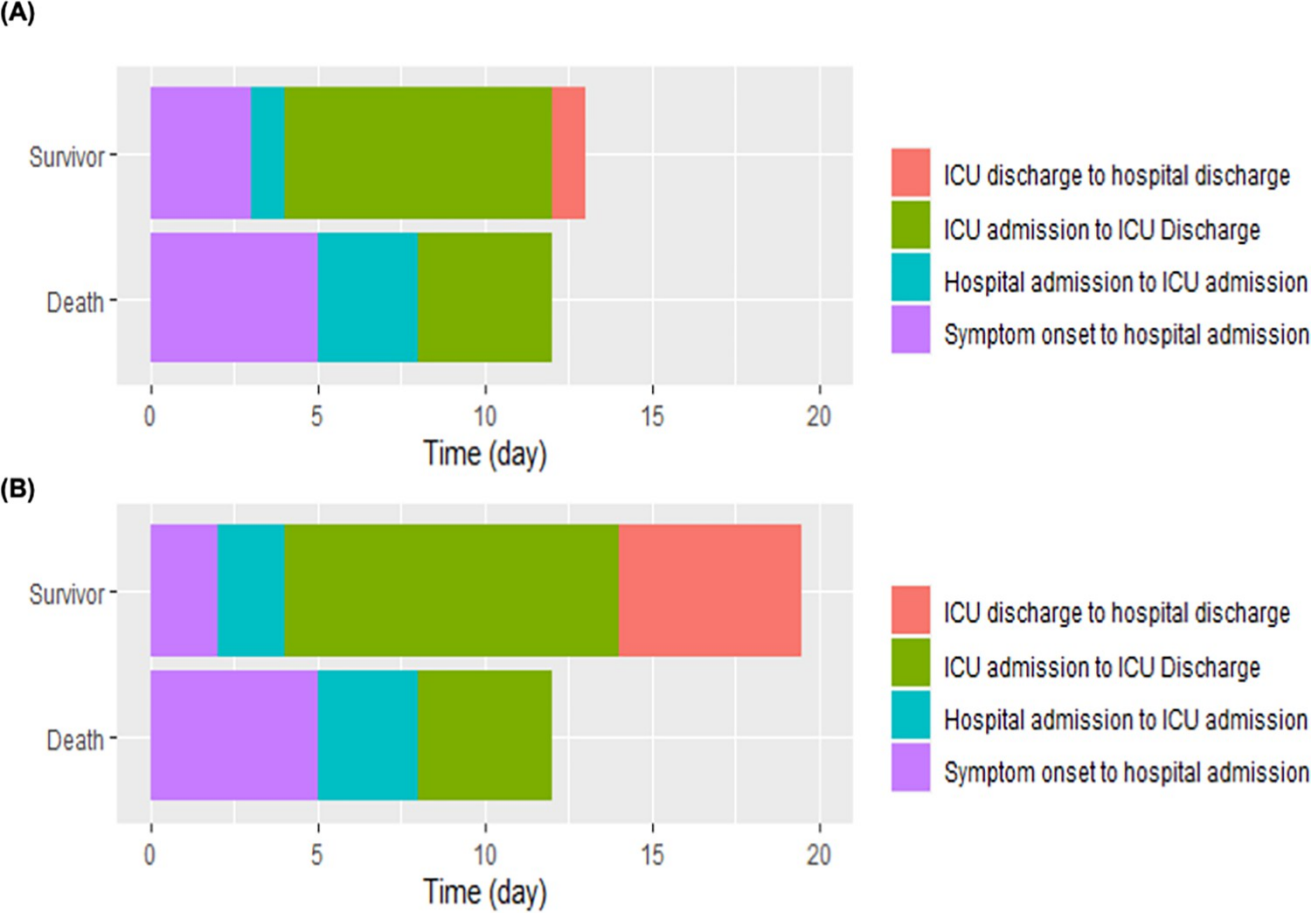
Differences in the time course from symptom onset between survivors and deaths. (A) Overall patients, (B) Patients who underwent mechanical ventilation during their ICU stays.

Intervals between symptom onset and hospital admission in surviving and fatal cases were 3 days [Inter Quartile Range (IQR); 1–7] and 5 days [IQR 3–7], respectively (Fig 2 and S1 Table). Hospital admissions to ICU admissions in the respective groups lasted for 1 day [IQR 1–3] and 3 days [IQR 1–5], respectively, while periods between the symptom onset and ICU admission were 4 days [IQR; 3–10] and 8 days [IQR 5–12]. These trends in the time course of COVID-19 infection also correspond to those in mechanically ventilated patients. Patients with unknown outcome data tended to have results more in keeping with survivors (S1 Fig).

### Baseline characteristics

Distributions of age, gender, and BMI were similar between both groups. Patients who died tended to have more comorbidities and symptoms associated with COVID-19 infection at the time of hospital admission. The number of the patients who had multiple symptoms of dyspnea, fever, cough, and fatigue/malaise simultaneously were 26 (14%) in the group of survivors and 98 (25%) in the group of deaths. These are same trends in the patients with mechanical ventilation except for age and gender. Among those who underwent mechanical ventilation, there were a significant difference in age (p < 0.001) and gender (p = 0.036) between the survivors and deceased patients (Table 1).

Changes of vital signs in the first 7 days of their ICU stay

**Fig 3:**
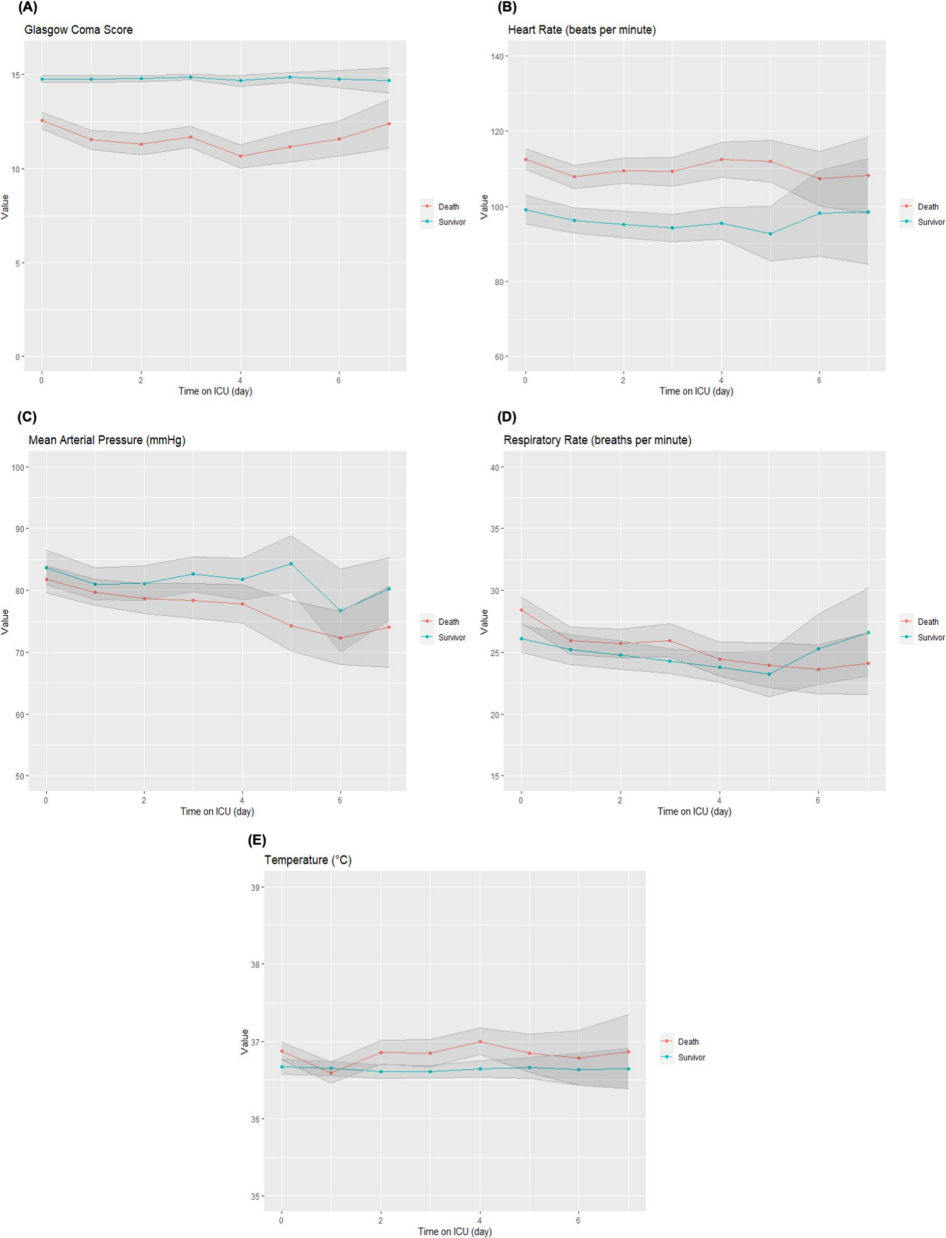
Changes of vital signs in the first week of ICU stay stratified by outcome, with 95% confidence intervals depicted by shaded regions. (A) Glasgow Coma Scale, (B) Heart Rate, (C) Mean Arterial Pressure, (D) Respiratory Rate, (E) Temperature; ICU intensive care unit.

Fatal cases showed decreasing trend of mean arterial pressure and respiratory rate during ICU stay, whereas temperature and heart rate remained comparable during the study period. Consciousness of fatal cases in GCS deteriorated to its lowest in the 4^th^ week then improved by the end of the study period, despite remaining lower than that of survivor group throughout ICU care, as in the case of mean arterial pressure (Fig 3).

### Treatments provided to the patients during ICU stay

The majority of the patients in both groups received oxygen therapy, numbering 81% and 88% in survivor and death groups respectively. High flow nasal canula, invasive mechanical ventilation, and vasopressors were administered in 47%, 81%, and 76% of death group respectively, higher than the 14%, 15%, and 21% administered in survivor group, while tracheostomy, ECMO, prone positioning, and nitric oxide were seldom applied in both groups. Renal replacement therapy, which was uncommon in both groups, were delivered in higher proportion of death group, namely, 15% as compared to 2.2% in survivor group. The most commonly used antivirus was oseltamivir, administered to more than half of all patients in death and survivor group, numbering 51% and 57% respectively, followed by remdesivir which was administered to the respective 8.7% and 5.5%, whereas other antivirals were hardly used in both groups. Hydroxychloroquine was used in 22% of both groups, while anti-interleukin 6 and convalescent plasma were not used in all but one subject in death group. A considerable percentage of death group, namely 11.3%, was treated with corticosteroid of varying types and doses, as compared to the 1.7% in survivor group. Anti-coagulation agents were not used as either prophylaxis or treatment in both groups (Table 2).

### Changes in laboratory parameters in the first 7 days of ICU stay

**Fig 4:**
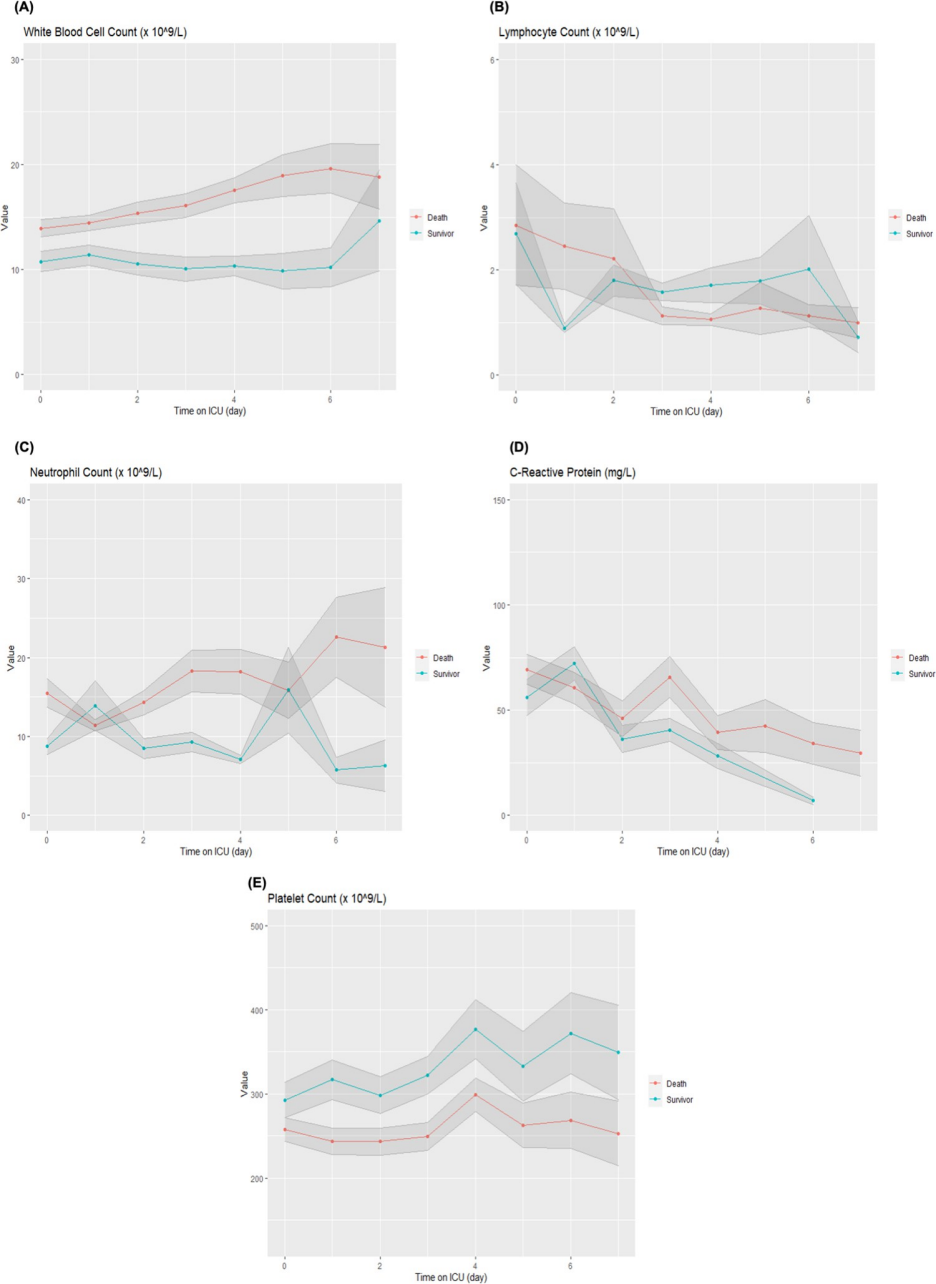
Changes of laboratory parameters in the first week of ICU stay, stratified by outcome with 95% confidence intervals depicted by shaded regions. (A)White Blood Cell Count, (B) Lymphocyte Count, (C) Neutrophil Count, (D) C-Reactive Protein, (E) Platelet Count; ICU intensive care unit.

The number of white blood cells were higher in patients who died, largely representing neutrophils. There were no differences in the number of lymphocyte cells by outcome. The C-reactive protein was higher in patients who died after three days of ICU stay. The platelet count was consistently lower in the group of patients who died though both groups were continuously in the normal range of platelet count (Fig 4).

### Complications during ICU stay

Complications were more frequent during the ICU stay for patients that died, such as bacterial infection as pneumoniae or bacteremia, pneumothorax, myocardial arrhythmia, seizure, stroke, pulmonary embolism, deep vein thrombosis, acute renal injury, gastrointestinal hemorrhage, and hyper or hypo glycaemia. Only congestive heart failure was more frequent in survivors than patients who died (Table 3).

### Other outcomes

Days from hospital admission to initiate antibiotics treatment was similar in both groups, while the group of death received fewer days of corticosteroid treatment regardless of presence of mechanical ventilation. The length of ICU and hospital stay, and mechanical ventilation period, are shown in Table 4. The primary cause of death was respiratory failure, followed by multi-organ failure and septic shock.

## Discussion

The government of Indonesia mandates all individuals to be covered by Jaminan Kesehatan Nasional (JKN), the country’s national health insurance program managed by Social Security Administering Body of Health (BPJS-K), a separate institution from the Ministry of Health [15]. During the COVID-19 pandemics, several policies were made by Indonesian government such as Large-Scale Social Restrictions Policy (Pembatasan Sosial Berskala Besar/PSBB) and The Policy for Enforcement of Restrictions on Community Activities (Pemberlakuan Pembatasan Kegiatan Masyarakat/PPKM) [16]. Pandemic-related health spending is covered by the government (excluded from JKN).

Patient mortality was consistently high over the first year of the pandemic in Indonesia, especially in patients who required mechanical ventilation during their ICU stay. The ICU patients who died frequently were apparently delayed in their admissions to hospitals and ICUs, despite reporting more symptoms at the time of hospital admissions. Less consciousness and higher heart rate for the entire first seven days of ICU stays were common in the ICU patients who died. They frequently received vasopressors and renal replacement therapy and experienced complications during their ICU stay, compared to the patients who survived. Many of the relevant therapies identified in early literature, such as prone positioning, ECMO, and neuromuscular blockade, were scarcely applied in both patients who survived and died.

This study demonstrated high patient mortality over the first year of the COVID-19 pandemic in Indonesia. Even though some decreasing trends were seen in the mortality of patients overall as the recent papers showed [17,18], the mortality of patients receiving mechanical ventilation was consistently high, around 90% over most of the first year in this study.

There are potential explanations for the high mortality. First, the patients who died were comparatively delayed in admitting to hospitals and ICUs. The higher number of symptoms on admission could also indicate this represents delayed presentation to hospital [19]. As previously studied, delayed presentation is a predictor of worse outcomes including death [20–22]. Alaa, et al found that each additional day between onset of symptoms and healthcare admission was associated with 1% increase in mortality risk (HR 1.01; p <0.005) [23]. Hospital admission time was associated with mortality in COVID-19 patients, thus showing the importance of utilizing strategies for timely hospital admission and providing easier access to healthcare. In an overwhelmed healthcare service, effective resource allocation, especially for ICU, which requires qualified personnel and advanced equipment is crucial. In low resource settings, patients may be less likely to immediately seek health care due to lack of awareness, access, or financial constraints [24]. These findings indicate that the appropriate monitoring outside the medical hospital and optimized rapid transfer systems should be managed in collaboration with the local health care system. The geographical features of Indonesia which consists of over 17,000 isolated islands could also potentially impede the immediate transfer of patients, resulting in delays for patients to reach hospitals. As the previous study has shown, the transfer of patients with COVID-19 infection requires strict infection control with personal protective equipment [25,26]. This could be conducted safely but matured local transport system is necessary [27,28]. Lack of suitable transport, prolonged travel time to health provider, and finances could be a barrier to appropriate healthcare service, especially in low-and middle-income countries (LMIC) [29].

Second, in this study, evidence supported approaches including HFNC before intubation [30,31], ECMO [32–35], prone positioning [26–36], and use of neuromuscular blockade [37,38] were not commonly performed. Only less than 10% of the patients received prone positioning which is now an established treatment for COVID-19 induced ARDS [39,40]. Proning was provided to over 50% of patients with COVID-19 infection in other papers from the same time [39]. ECMO is also recommended as a last-line therapy for ARDS and around 5–10% of patients with COVID-19 infection are epidemiologically eligible for that approach [41]. In this study, HFNC and neuromuscular blockade were provided slightly more frequently to patients who died. Given the delay of admission to hospitals and ICUs where medical interventions can be provided, the introduction of these evidence based approach might have also been late. Prone positioning and ECMO are treatments that are relatively resource intensive. During the first pandemic, many hospitals were forced to cope with limited resources and inexperienced staff in an overwhelmed ICU. Many papers published during the first phase of the pandemic showed high mortality of COVID-19 infection, around 50–80% in the mechanically ventilated patients [42,43]. For mechanically ventilated COVID-19 patients, Namendys-Silva, et al in Mexico found an overally in-hospital mortality of 73.7% [44], while King, et al in America found 42.7% mortality [45]. A study on expanded ICU in New York, when the patient load surpassed traditional ICU capacity, had 61% mortality within 28 days after intubation [46]. In China, Wang et al found 79.4% deaths at/before 28 days in COVID-19 patients treated with mechanical ventilation (noninvasive or invasive), and the death toll in patients with invasive ventilation was 97% [47]. Given extreme high mortality, it is noteworthy that the impact of the pandemic might be larger in lower income countries which generally suffer from limited resource availability [6]. It is also worth noting that the exclusion of patients with incomplete data may skew these mortality results. It is highly likely that for periods where the mortality rate approached 100%, there were patients that survived that were lost to follow-up either due to interhospital transfer or due to movement to a rehabilitation or community care centre. Furthermore, Indonesia is geographically isolated from other countries and is composed of multiple islands. Resource distribution and transport of the patient to the right hospitals even inside the country are serious issues to be addressed [48,49]. During the early phase of pandemic, the national COVID-19 guideline was still developing along with evidence based COVID-19 therapies, and certain treatment protocols like HFNC, ECMO and prone positioning have been underutilized. The management options in the national guideline were being updated in response to new evidence. The guideline has reached its 4^th^ edition as of today [14].

Another potential explanation of the high mortality is frequent complications observed in the patients that died during their ICU stay. For example, acute renal failure was found almost twice as frequently in patients that died. As previous papers have shown, acute renal failure during ICU stay is one of the independent factors associated with mortality [50,51]. The complication often occur along the course of COVID-19 hospitalization and is associated with increased illness severity, duration of stay, and poor prognosis [52]. Elkholi, et al found that AKI occurred in 65.1% of mechanically ventilated COVID-19 patients, and it was associated with significantly higher mortality [53]. Given that the data is from a registry and the study is retrospective, determining the cause-effect relationship is outside the scope of our study, and further studies are need to understand this aspect. Even more interestingly, the patients received renal replacement therapy was less than 20% even though around half of the patients who died faced acute renal failure. This might indicate that the introduction renal replacement therapy was delayed or not performed appropriately [54]. The implementation of the treatment in right time has been suggested to be a key element to maximize the effect of a medical intervention on outcomes [55,56]. Further research regarding the administration and timing of renal replacement therapy in Indonesian COVID-19 patients in Indonesia is needed. There was a relatively higher congestive heart failure incidence in survivors, which could be caused by sicker patients dying before heart failure could occur, creating a survival bias.

Several limitations were acknowledged. First, this study design of a prospective observational study could not show the causal relationship. To evaluate the causal inference, a robust analytic method might need to be applied. Furthermore, there were missing data in the primary outcome, survival or death, which could potentially result in large selection bias. Second, most of the patients enrolled in this study were those admitted to ICU before the first wave of COVID-19 pandemic in Indonesia. Therefore, the characteristics and outcomes of ICU patients during the first wave could be different from our results because of the overwhelmingness against the hospital and ICU capacity. Third, the results could limit the generalizability since this study focused only the patients with COVID-19 infection from Indonesian large tertiary hospitals included in the ECMOCARD registry, limiting the representation of data from other smaller or rural hospitals across the region. It is necessary to allow for the differences in culture, financial, and political policy between the countries when the results are interpreted, especially outside of Indonesia. Finally, the following unmeasured and essential factors associated with the mortality have not investigated in this study: respiratory mechanical function during receiving mechanical ventilation, spontaneous awakening and breathing trials, delirium, use of sedation agents, and management of fluid strategy. In invasively ventilated COVID-19 patients with ARDS, higher cumulative fluid balance was found to be associated with longer duration of ventilation [57], while other studies found that early short course neuromuscular blocking agents (NMBA) increased the risk of 28-day mortality by 2.2 times [58] and did not significantly improve 90-day mortality [59]. Compared to ARDS from other etiologies, COVID-19 ARDS patients received larger dose of hypnotics, which was associated with coma and higher in-hospital mortality [60]. Further research are needed to address these limitations in order to have a deeper understanding of the experience and outcomes of COVID-19 ICU patients in Indonesia. Another limitation of our study is the incomplete results of PCR. This might be due to the inconsistent diagnostic approach during the first wave, whereas Indonesian hospitals used antibody test and PCR tests were limited during the first wave. Vaccination for COVID-19 data is also an acknowledged limitation, since vaccination for COVID-19 enhance the clinical outcome and the severity, hence increasing the survival rate [61]. Our database did not mention COVID-19 vaccination due to the late start of the vaccination (in January 2021) [62]. Further study might be needed in this area.

## Conclusions

For the first year of the pandemic in Indonesia, the mortality rate of patients with COVID-19 infection was extremely high, especially in patients who underwent mechanical ventilation during ICU stays, consistently showing 90%, compared to 50–80% in other studies. Delayed admission and a great burden for the availability of health resources in Indonesia during pandemic may impact on the high mortality in this cohort, thus showing the importance of efficient public health measures and enhancing health infrastructure for future pandemic response. Other key areas that may need to be addressed were the lack of evidence-based approaches, particularly the prone position application, which could be evaluated between health policy makers and medical associations for a more standardized national health care service supported by evidence-based guideline updates.

## Supporting information

S1 Fig Differences in the time course from symptom onset between survivors and deaths with additional bar for patients with unknown final outcome. (DOCX)

S1 Table Details of the differences in the time course from symptom onset between survivors and deaths. (DOCX)
