# Supplementary material for: Characteristics and outcomes of patients with severe COVID-19 in Indonesia: Lessons from the first wave
Source: PLoS One. 2023 Sep 25;18(9):e0290964. doi: 10.1371/journal.pone.0290964 (PMC10519602; doi:10.1371/journal.pone.0290964)
Supplement: S1 Fig — (DOCX) [file pone.0290964.s001.docx]

**S1**. **Differences in the time course from symptom onset between survivors and deaths with additional bar for patients with unknown final outcome.**

**(A)**


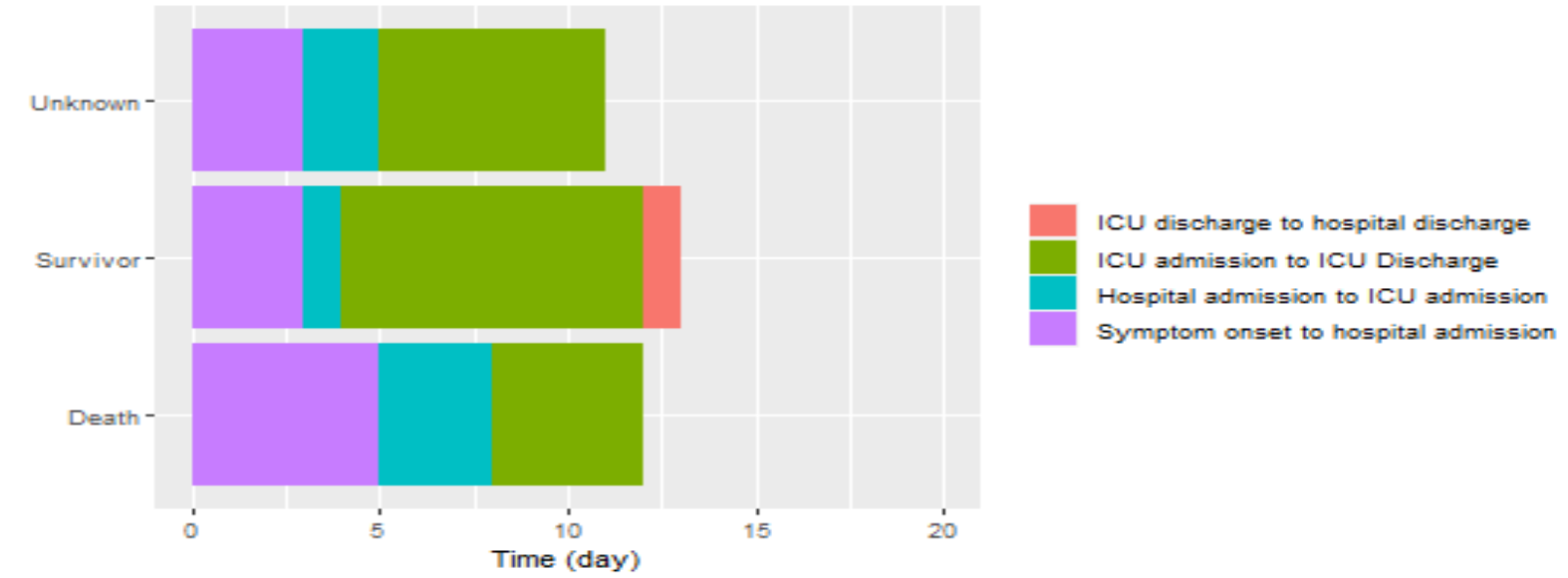


**(B)**


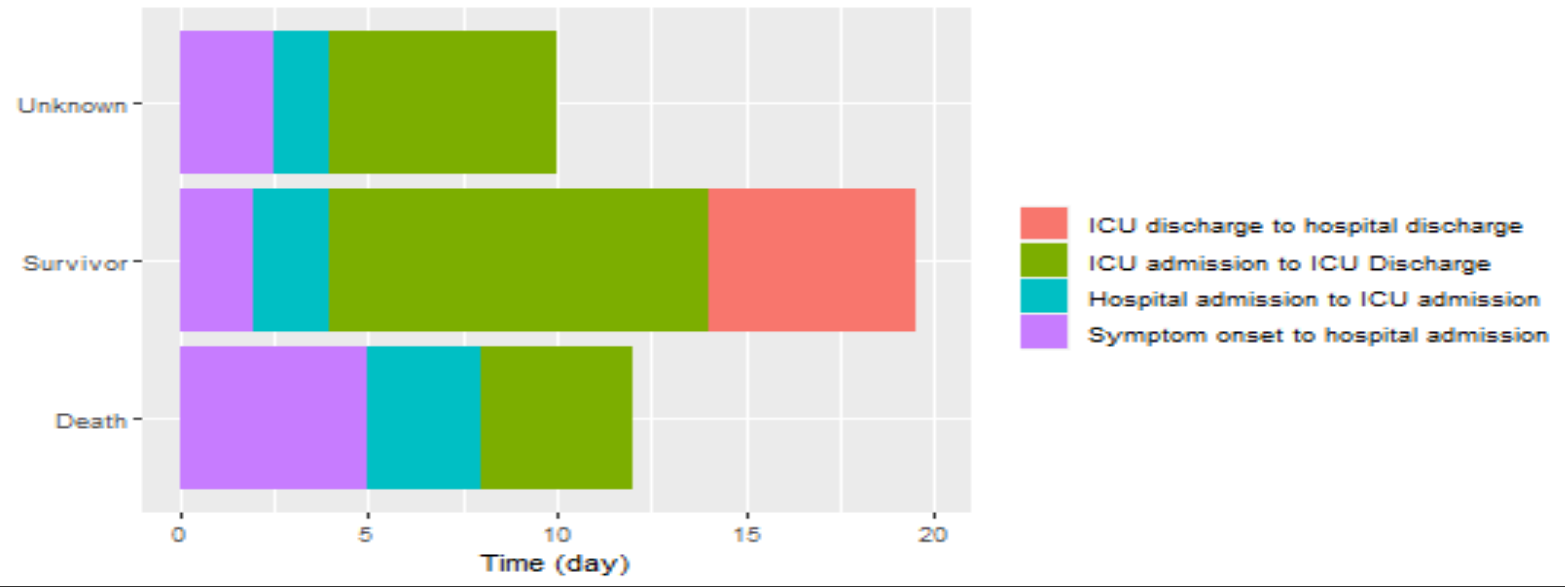


(A) Overall patients, (B) Patients who underwent mechanical ventilation during their ICU stays
