# Supplementary material for: Characteristics and outcomes of patients with severe COVID-19 in Indonesia: Lessons from the first wave
Source: PLoS One. 2023 Sep 25;18(9):e0290964. doi: 10.1371/journal.pone.0290964 (PMC10519602; doi:10.1371/journal.pone.0290964)
Supplement: S1 Table — (DOCX) [file pone.0290964.s002.docx]

**Supplemental Table 1**. Details of the differences in the time course from symptom onset between survivors and deaths

| Variable | All patients | Survivors | Deaths | Mechanical Ventilation | |
| --- | --- | --- | --- | --- | --- |
|  |  |  |  | Survivors | Deaths |
| Time (days), median (IQR) |  |  |  |  |  |
| From symptom onset to hospital admission | 4 (2-7) | 3 (1-7) | 5 (3-7) | 2 (1-7) | 5 (3-7) |
| From hospital admission to ICU admission | 2 (1-4) | 1 (1-3) | 3 (1-5) | 2 (1-3) | 3 (1-5) |
| From symptom onset to ICU admission | 8 (4-12) | 4 (3-10) | 8 (5-12) | 4 (3-9) | 8 (5-12) |
| From ICU admission to ICU discharge | 5 (2-10) | 8 (5-14) | 4 (2-8) | 10 (5-20) | 4 (2-9) |
| From ICU discharge to hospital discharge or death | 0 (0-0) | 1 (1-6) | 8 (4-14) | 5 (0-7) | 8 (5-14) |

IQR interquartile range, ICU intensive care unit
